# Recommender System Based on Collaborative Filtering for Personalized Dietary Advice: A Cross-Sectional Analysis of the ELSA-Brasil Study

**DOI:** 10.3390/ijerph192214934

**Published:** 2022-11-13

**Authors:** Vanderlei Carneiro Silva, Bartira Gorgulho, Dirce Maria Marchioni, Sheila Maria Alvim, Luana Giatti, Tânia Aparecida de Araujo, Angelica Castilho Alonso, Itamar de Souza Santos, Paulo Andrade Lotufo, Isabela Martins Benseñor

**Affiliations:** 1 Department of Epidemiology, School of Public Health, University of São Paulo, São Paulo 01246-904, Brazil; 2 Center of Clinical and Epidemiological Research, University Hospital, University of São Paulo, São Paulo 05508-000, Brazil; 3 Department of Food and Nutrition, School of Nutrition, Federal University of Mato Grosso, Cuiaba 78060-900, Brazil; 4 Department of Nutrition, School of Public Health, University of São Paulo, São Paulo 01246-904, Brazil; 5 Institute of Collective Health, Federal University of Bahia, Salvador 40110-040, Brazil; 6 Department of Social and Preventive Medicine, Faculty of Medicine & Clinical Hospital, Federal University of Minas Gerais, Belo Horizonte 30130-100, Brazil; 7 Laboratory of the Study of Movement, Faculty of Medicine, University of São Paulo, São Paulo 05403-010, Brazil

**Keywords:** recommender system, collaborative filtering, diet, dietary advice, algorithms

## Abstract

This study aimed to predict dietary recommendations and compare the performance of algorithms based on collaborative filtering for making predictions of personalized dietary recommendations. We analyzed the baseline cross-sectional data (2008–2010) of 12,667 participants of the Brazilian Longitudinal Study of Adult Health (ELSA-Brasil). The participants were public employees of teaching and research institutions, aged 35–74 years, and 59% female. A semiquantitative Food Frequency Questionnaire (FFQ) was used for dietary assessment. The predictions of dietary recommendations were based on two machine learning (ML) algorithms—user-based collaborative filtering (UBCF) and item-based collaborative filtering (IBCF). The ML algorithms had similar precision (88–91%). The error metrics were lower for UBCF than for IBCF: with a root mean square error (RMSE) of 1.49 vs. 1.67 and a mean square error (MSE) of 2.21 vs. 2.78. Although all food groups were used as input in the system, the items eligible as recommendations included whole cereals, tubers and roots, beans and other legumes, oilseeds, fruits, vegetables, white meats and fish, and low-fat dairy products and milk. The algorithms’ performances were similar in making predictions for dietary recommendations. The models presented can provide support for health professionals in interventions that promote healthier habits and improve adherence to this personalized dietary advice.

## 1. Introduction

The adoption of a healthy lifestyle is recognized as an important component of chronic disease prevention and management [1]. Diabetes, cardiovascular diseases, cancer, and obesity represent a major burden in health care systems worldwide. Among the main factors, physical exercise, reduction in alcohol consumption, smoking cessation, and a healthy diet are essential to reduce the risk of these diseases. However, adherence to these recommended behavior changes is generally extremely low. Nonadherence rates to the treatment of chronic diseases are estimated to be between 50 and 80% [2].

Over the years, health management on a personal level has evolved and been supported by technology [3]. Recommender systems are widely used to help users find new items or services, such as books, music, transportation, and even people [4]. Among the existing recommender systems, collaborative filtering (CF) has gained significant success, and two approaches are common to predict ratings, that is, the preference for a service or product measured through a scale that expresses the degree of like or dislike of a person [5,6]. User-based CF methods recognize people related to the analyzed individual and predict the rating to be the average ratings of similar people. In the same way, item-based CF identifies items related to the demanded product (for example, a movie) and predicts the rating to be the average of the ratings of similar products [6].

In the field of health, recommender systems have been designed with a large amount of data to assist experts in making clinical decisions and making treatment recommendations [7,8]. Among health recommender systems, there are applications for medication prescription [9], support for smoking cessation [10], and depression and anxiety management [11]. Recommender systems are still not widely used in support of dietary advice, which indicates a research opportunity to fill this gap [12,13]. The system could use dietary intake data as input and provide as an output a set of recommendations most likely to be adopted by people [14,15]. Effective dietary advice that increases adherence to healthier dietary intake, for example, is always a great challenge [16].

Evidence has demonstrated that many people face the problem of making healthier food decisions that will impact their health and risk of noncommunicable diseases [17,18]. The use of dietary recommender systems could significantly contribute to health care and guide professionals to identify food groups that are more likely to lead to adherence based on specific sociodemographic and clinical profiles. Therefore, given the success of applications in the diagnostic and prescriptive medicine fields, there is a felt need to attempt and validate a recommender in the public health and nutrition education fields. This study aimed to predict a set of dietary recommendations based on current guidelines, with a focus on healthy diets to which the user could adhere more easily, and compare the performance of two machine learning algorithms, user-based and item-based, for making personalized dietary recommendations.

## 2. Materials and Methods

### 2.1. Participant Recruitment

ELSA-Brasil is a multicenter prospective cohort study designed to investigate the incidence of cardiovascular diseases and diabetes and their biological, environmental, occupational, and social determinants. The participants were 15,105 active and retired civil servants, male and female, recruited from teaching and research institutes in the following six Brazilian cities: Salvador, Belo Horizonte, Vitória, Rio de Janeiro, São Paulo, and Porto Alegre [19,20]. Active or retired employees aged 35 to 74 years who answered the Food Frequency Questionnaire (FFQ) were eligible to participate. Cross-sectional data from the baseline examination (2008–2010) were analyzed. The exclusion criteria were as follows: the intention to leave the institution, current or recent pregnancy within the prior four months, severe cognitive or communication difficulty, and, if retired, residence outside the research center metropolitan region. After recruitment, the participants were interviewed at the work facility (Phase 1) and scheduled a date to visit the research center to undergo several exams, such as anthropometrics, blood pressure measurements, and electrocardiograms (Phase 2). Of the total sample, we excluded n = 2438 (16%) participants with an implausible daily energy intake of less than 500 or greater than 4000 Kcal/day [21]. The final sample was composed of 12,667 public employees, of whom 5217 (41%) were men and 7450 (59%) were women. This study was performed according to the guidelines suggested by the Declaration of Helsinki, and the study protocol was reviewed and approved by the Ethics Committee of the School of Public Health of the University of São Paulo under number 2.566.286. After explaining the purpose of the survey informed consent was taken from the study participants who were willing to participate in the study. The data are reported according to the STROBE guidelines for cross-sectional studies.

### 2.2. Dietary Assessment

Dietary assessment was performed using a semiquantitative Food Frequency Questionnaire (FFQ) developed for the ELSA-Brasil study. The FFQ presents a list of 114 food items and was based on a previously validated questionnaire [22]. The study participants were asked by trained interviewers about their frequency and amount of consumption of each food over the 12-month period preceding the interview. The daily intake was quantified by the number of servings consumed per day × weight (standard portion in grams) × frequency of consumption × nutritional composition of the food serving. Nutrition Data System for Research (NDSR) software (University of Minnesota, Minneapolis, MN, USA, 2010) was used to determine the nutritional composition of the foods and preparations and daily energy intake in kilocalories. Details about the elaboration [22] and validation of the questionnaire [23] can be obtained in previous publications.

**Figure 1:**
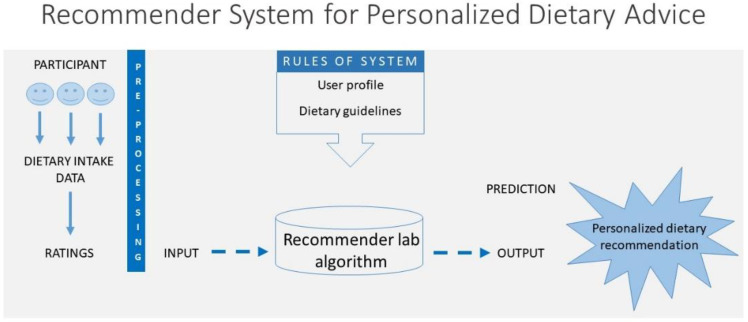
The architecture of the dietary recommender system for ELSA-Brasil study participants.

For the analysis of algorithms in the recommender system, the items from the FFQ (114 foods or preparations) were collapsed into 21 groups by food preparation or nutritional characteristics: refined cereals; whole cereals; tubers and roots; breads; confectionery; beans and other legumes; oilseeds; fruits; vegetables; red meats; white meats and fish; processed meats; eggs; high-fat dairy products and milk; low-fat dairy products and milk; oils and fats; pasta; salted snacks; juices and other beverages; soft drinks; and alcoholic beverages (Supplementary Materials, Table S1). Although all foods were used as input in the recommender system, the list of items eligible as recommendations included 8 of the 21 food groups: whole cereals; tubers and roots; beans and other legumes; oilseeds; fruits; vegetables; white meats and fish; low-fat dairy products and milk. These food groups were fixed based on the current Brazilian dietary guidelines with a focus on diets with a recognized impact on health promotion and a reduction in the risk of chronic diseases [24]. In contrast to many common recommender systems (e.g., online shopping based on previous purchases), the food items that users like and consume the most are not necessarily the healthiest [25]. Thus, the recommendations were based on the system’s ability to provide suggestions for healthy foods and an emphasis on items to which the participant could adhere (Figure 1).

### 2.3. Sociodemographic and Clinical Characteristics

The participants were required to visit the research center for clinical tests and interviews, and their clinical, demographic, dietary, and behavioral data were collected [20]. The features collected were included in the analysis to describe our sample and compare the subsets derived in the train and test stages of the models which were: sex (male vs. female), age (years), education level (elementary (or lower), high school, college), retirement (no vs. yes), self-reported race/ethnicity (white, brown, black, other (Asian, Indigenous)), per capita income in USD categorized in terciles, using USD 1.00 = BRL 2.00 as the approximate baseline examination exchange rate, living alone or with another person (with another person vs. alone), marital status (not married vs. married), smoking habit (never, ex-smoker, current smoker), physical activity (sedentary, insufficiently active, active (using the leisure time section of the long version of the International Physical Activity Questionnaire)), health self-assessment (good, regular, bad), and location of the research center (Salvador, Belo Horizonte, Vitória, Rio de Janeiro, São Paulo, or Porto Alegre).

Weight and height measurements were obtained with the participant wearing light clothes and without shoes. The body weight was measured to the nearest 0.1 kg with a calibrated balance (Toledo 2096PP), and height was measured with a vertical stadiometer (Seca-SE-216) to the nearest 0.1 cm. The body mass index (BMI) was calculated by dividing the weight in kilograms by the height in meters squared (kg/m^2^). The waist circumference was measured with a tape measure to the nearest 0.1 cm around the midpoint between the inferior costal border and the iliac crest, while the hip circumference was measured at the point of greatest circumference in the gluteal region. The waist-to-hip ratio (WHR) was calculated by dividing the waist size by the hip size in centimeters.

Blood pressure (BP) was measured using a validated (Omron HEM 705CPINT) oscillometer. Three measurements were taken at one-minute intervals, and the mean of the two latter blood pressure measurements was considered the value for defining hypertension, defined as systolic blood pressure at ≥140 mm Hg, diastolic blood pressure at ≥90 mm Hg, or verified treatment with antihypertensive drugs during the last two weeks.

All laboratory parameters were measured in blood samples collected in the local investigation centers, after a mean 12-h fasting period (minimum of 10 h, and maximum of 14 h). Triglyceride levels were measured by using the colorimetric method containing glycerophosphate and peroxidase. LDL levels were estimated by using the Friedewald formula, and, when the TG levels were higher than 400 mg/dL, a homogeneous enzymatic colorimetric assay without precipitation was used. HDL levels were measured using a homogeneous enzymatic colorimetric assay without precipitation. Glucose was measured by a hexokinase method (ADVIA 1200 Chemistry; Siemens). Glycated hemoglobin (Hb A1c) was measured by using HPLC (Bio-Rad D-10 Dual Program Laboratories). Dyslipidemia was defined as LDL cholesterol ≥ 130 mg/dL or the use of medication to treat dyslipidemia. Diabetes was defined as a previous diagnosis of diabetes, the use of medication to treat diabetes, fasting plasma glucose ≥ 126 mg/dL, 2-h plasma glucose ≥ 200 mg/dL, or HbA_1C_ ≥ 6.5%. Cardiovascular disease was defined as self-reported prior myocardial infarction, stroke, or revascularization.

### 2.4. Statistical Analysis

The continuous variables are presented as the medians and interquartile ranges, and the categorical variables are presented as frequencies. Comparisons of the values for the continuous variables by data set (i.e., train or test) were performed using a Mann–Whitney test. Associations among the categorical variables were tested through the chi-square test. The results were considered significant at *p* < 0.05. The analyses were performed using R software, version 4.0.2.

### 2.5. Recommender System

Typically, a person’s dietary intake is assessed and then used as input for decision-making to provide feedback to the person [25]. This concept was implemented in the architecture of the system, as shown in Figure 1. The recommender system was designed to promote healthier dietary choices, with a recognized impact on risk reduction and the prevention of chronic diseases [26,27].

All analyses were performed using R software, version 4.0.2. The data set was mapped in the form of a rating matrix by the creator function recommender on the recommender lab package [28]. Each row indicates a study participant, and the column indicates a food group. The dietary intake data were categorized into quintiles and transformed into a scale between 1 and 5 (ratings); that is, the recommender system used dietary intakes that were transformed into ratings [6]. The absence of consumption represented foods that could be used for the recommendation. If a participant had a missing value in the data set, for example, absence of the consumption of beans, these foods could be recommended. The recommender system predicts the ratings (preferences) that a user would give to an item [6]. Thus, missing values were replaced by estimated ratings.

Before recommendation, the system identified participants who shared the same food intake. The Pearson correlation coefficient was used as a measure of the similarity of the participants’ daily usual dietary intakes. The recommendations were based on the calculations of similarity among peer individuals with a similar diet. For every participant, the algorithm identified the K-most similar [28]. The premise is that people who agree on the intake profile for some foods typically also agree on the rating for other items. The ratings for an individual can be predicted by first finding a neighborhood of similar users and then aggregating the ratings of these users to form a prediction [6,29].

User- and item-based CF algorithms were used, and a range maximum of 5 recommendations was fixed to avoid unspecified and very extensive recommendations [5]. The recommender system was designed to predict a list of the top-N dietary recommendations. Therefore, to create a top-N recommendation list, the food items were ordered by their predicted rating. The participants were randomly divided into two subsets. The first was used for training (70%), and the second was used for testing (30%). The train users were used to learn the recommender model and suggest food groups, whereas the test users were used to evaluate the recommendations. Some foods were withheld from the testing base before the recommendations were created. It was assumed that if a recommender algorithm performed better in predicting the withheld items, it would also perform better in finding good recommendations for unknown items [28].

The prediction function was used to predict the ratings of unknown items by the algorithm in the test data set. The difference between the finally predicted value and the actual correct answer was defined as an error value. The function “calc Prediction Accuracy” was used to calculate the accuracy of the predictions.

## 3. Results

### 3.1. Architecture of the Recommender System

The recommender system was designed to promote healthier dietary choices. The dietary intake was assessed and then used as input for decision-making to provide feedback. The architecture of the system is shown in Figure 1.

### 3.2. Descriptive Analyses

Table 1 shows the characteristics of the study population. There were no statistically significant differences between the two groups according to the sociodemographic and health data. The most common sample consisted of women (59%) who had a median age of 52 years old (IQR 45–59), a high education level, and who were active workers, white, and not single. They reported being mostly nonsmokers and sedentary and self-reported their health as good. Among the health and clinical characteristics, the median body mass index was 26.3 kg/m^2^ (IQR 23.7–29.5), and the waist-to-hip ratio was 0.9 (IQR 0.8–1.0). The frequency of dyslipidemia was 59%, hypertension 36%, diabetes 16%, and cardiovascular disease 4%.

### 3.3. Food Groups and Items Eligible as Recommendations

Table S1 in the Supplementary Materials shows the food groups and list of items eligible as recommendations. All groups were analyzed by the recommender system, but the eligible recommendations were as follows: whole cereals; tubers and roots; beans and other legumes; oilseeds; fruits; vegetables; white meats and fish; low-fat dairy products and milk.

### 3.4. Evaluation of Predictions

Table 2 shows the error metrics by model. The root mean square error (RMSE), mean squared error (MSE), and mean absolute error (MAE) were used to compute the deviation of the prediction from the true value. Compared to item-based collaborative filtering (IBCF), user-based collaborative filtering (UBCF) had a lower error rate—RMSE: 1.49 vs. 1.67; MSE: 2.21 vs. 2.78; MAE: 1.26 vs. 1.40.

### 3.5. Confusion Matrix

Table 3 compares the predictive performance between the models by the k nearest neighbors. UBCF and IBCF showed similar performances, with precision between 0.88 and 0.91 and a plateau when k = 10 was used. The precision refers to the percentage of recommended food items with intake, while recall refers to the percentage of intake food items that have been recommended. Other metrics are also presented. There were no differences between the two models.

### 3.6. ROC Curve and Precision–Recall

**Figure 2:**
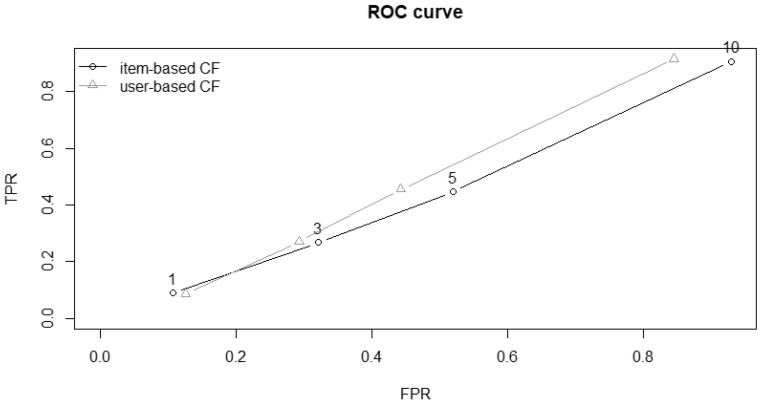
ROC curve, a possible way to compare the efficiency of two systems by comparing the size of the area under the curve, where a larger area indicates better performance. The default plot of the ROC curve plots the true positive rate (TPR) against the false positive rate (FPR).

**Figure 3:**
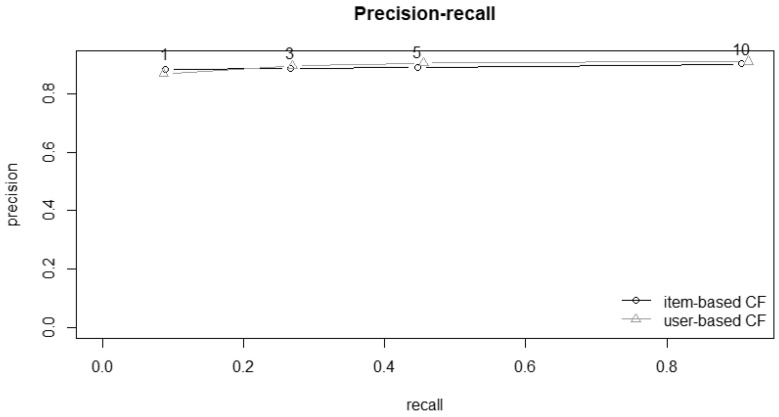
Comparison of precision–recall curves for item- and user-based recommender methods; precision represents correctly recommended items divided by total recommended items; recall represents correctly recommended items divided by total useful recommendations.

Figure 2 and Figure 3 show the ROC curves and plots for precision–recall. Both confirm the similar performances between the two algorithms.

## 4. Discussion

The results show that there were no differences between the user- and item-based collaborative filtering algorithms with regard to their performance. This finding confirms the hypothesis that a food recommender system can analyze individuals’ diet data and provide predictions of personalized dietary recommendations. The main contribution of this study is the presentation of a tool to predict dietary recommendations to which users are likely to adhere, which can be beneficial for groups of people to change their eating habits. In addition, health specialists can gain a better understanding of diet characteristics by obtaining more accurate models of their patients and promoting healthier habits [30].

Although previous studies have used different methods, they have also applied recommendation systems in the field of nutrition [13,25,31]. Chen et al. applied deep learning neural network models and compared different data sets from grocery products with accuracies of 72–84% [13]. The categorized grocery products were compared to their own group and recommended to the consumer. Norouzi et al. analyzed Iranian women and men (n = 30) and focused on the development of a food recommender system for managing diabetic patients’ nutrition [31]. The roulette wheel algorithm was used, and a snack with a higher ranking was recommended to the patient. The results showed that the system recommended various snacks according to the season (accuracy of 100%) and personal interest (accuracy of 90%) for diabetic patients. Our results are similar to those of previous studies, with accuracies between 88% and 91%.

The recommender system used dietary intake data that were transformed into ratings. The system locates peer users with a similar diet, and the foods with the highest rating predicted for an individual were recommended. Various statistical techniques, such as Euclidean distance, cosine similarity, and Pearson correlation, can be used to compute the similarities among users [32]. In our study, Pearson’s correlation coefficient was used to find the nearest neighborhoods. Thus, our recommender system, as shown in Figure 1, can deliver as an output an individualized list with suggestions for healthier food intakes that can be used as recommendations for a healthier diet. The prediction itself is an ordered list of those items in the study participant’s diet whose advice would be most likely to adhere to it.

Although recommendation systems are expanding in many areas, they are still underutilized to promote dietary changes, especially with a preventive nature. Moreover, previous systems have been proposed for specific health issues but lack focus on health and disease prevention. An advantage of their use is that personalized advice is more effective than general population-based recommendations for modifying health-related behavior in nutrition interventions [33]. Furthermore, knowledge about a healthy diet is not sufficient on its own to change eating behavior, but individualized feedback has been found to be associated with higher adherence to interventions to promote healthy lifestyles [34].

The results show very small differences in favor of user-based collaborative filtering, so the two approaches could be useful if applied in the context of health education. However, user-based CF may consider a patient’s social environment. This can be useful for the system to recommend healthy foods that are also part of the individual’s culture, that is, foods that are present in the diet of his or her peers. The technique was based on the concept that people who have an interest in a particular food may have similar interest in other foods [35].

Recommender systems should not replace the methods usually used for dietary assessment or the assistance of qualified health professionals. However, they can be used as a complementary approach as well as support for dietary advice [36]. The output delivered by the systems should be validated and combined with other information, such as socioeconomic aspects, health and clinical conditions, anthropometric and biochemical data, and the use of biomarkers, according to the objective and needs of each person [37].

The maximum number of recommendations was fixed to five, although other cutoffs could also be established. Extensive recommendations can discourage adherence, and dietary changes are more effective when they are adopted gradually [38]. Furthermore, while habits are consolidated, a new dietary assessment can be conducted. Recommendations that consider changes in the patient’s situation should be adapted over time.

Some limitations should also be addressed. The FFQ allows for the collection of participants’ usual intake regardless of intraindividual variability in addition to ranking people in consumption ranges. However, it has limitations similar to other assessment methods, especially with regard to not capturing details about the diet, such as tastes, preferences, negative or positive reactions to certain foods and preparations, restrictions, intolerance, allergies, or even main concerns about diet and health that are relevant to dietary advice. Therefore, recommendations should be interpreted in a holistic context, especially in the strata of the population with socioeconomic restrictions. The data analyzed refer to the baseline of the ELSA-Brasil study; however, health crises, such as the COVID-19 pandemic and changes in the world economy due to unpredictable events, can impact dietary choices and access to food that were not captured by our current recommender system. In addition, the data in this manuscript were from the 2008–2010 (baseline of the ELSA-Brasil study) and it was not possible to compare dietary choices with more current data in the context and objectives of the analysis presented. Another point is the limited generalizability of the results to populations with other characteristics and individuals with specific requirements.

This study has some strengths. The data analyzed were from a large sample of older and middle-aged adult individuals and incorporated a catalog of typical/regional foods. Although the sample consisted only of civil servants, it aggregated a mixed multiethnic population and captured non-isolated eating practices. The study helps to fill an important gap in the literature since there are few examples of dietary recommender systems that provide people with content to improve the quality of their diet. The engine could be used as a clinical decision support system. The FFQ was developed and validated in the study population. The participants were invited to attend a clinical research center for exams and clinical evaluations, which guaranteed a high standard of quality control for the data used in the study.

## 5. Conclusions

This work opens a discussion about the applications of automated intelligence systems in the field of nutrition. The algorithms evaluated the set of possible recommendations and highlighted those to which participants were most likely to adhere. Future work can assess whether the adherence to recommendations differs when an automated tool is used to support a human expert compared to interventions without the support of technological tools. Communication technologies provide new potential and offer several advantages, such as lowering costs and improving outcomes, by reaching a larger segment of the target population [39]. On the other hand, it is a consistent finding that human support is also necessary to ensure adherence (i.e., following the intervention protocol) and to increase the effects [40]. Therefore, technology does not replace health specialists but can represent benefits in more personalized health care.

## Figures and Tables

**Table 1:** Clinical and Socioeconomic characteristics of the study population, Brazilian Longitudinal Study of Adult Health (ELSA-Brasil), 2008–2010.

Variable	General		Train		Test		*p* Value ^9^
	n	%	n	%	n	%	
Study population	12,667	100.0	8866	100	3801	100	
Sex							
Male	5217	41.2	3665	41.3	1552	40.8	
Female	7450	58.8	5201	58.7	2249	59.2	0.596
Age (years) ^1^	52	45–59	52	45–59	52	45–59	0.850
Education level							
Elementary (or less)	1423	11.2	1022	11.5	401	10.5	
High school	4072	32.2	2829	31.9	1243	32.7	
College	7172	56.6	5015	56.6	2157	56.8	0.247
Retirement							
No	10,046	79.3	7012	79.1	3034	79.8	
Yes	2621	20.7	1854	20.9	767	20.2	0.351
Race/ethnicity							
White	6994	55.2	4887	55.1	2107	55.4	
Mixed	3379	26.7	2373	26.8	1006	26.5	
Black	1831	14.4	1281	14.4	550	14.5	
Others ^2^	463	3.7	325	3.7	138	3.6	0.986
Marital status							
Not single	8181	64.6	5700	64.3	2481	65.3	
Single	4486	35.4	3166	35.7	1320	34.7	0.290
Per capita income ^3^							
1° tercile	4225	33.4	2994	33.8	1231	32.4	
2° tercile	4492	35.5	3093	34.9	1399	36.8	
3° tercile	3950	31.2	2779	31.3	1171	30.8	0.103
Living alone							
No	11,043	87.2	1157	13.1	467	12.3	
Yes	1624	12.8	7709	86.9	3334	87.7	0.239
Smoking habit							
Never	7306	57.7	5137	57.9	2169	57.0	
Ex-smoker	3780	29.8	2643	29.8	1137	29.9	
Current smoker	1581	12.5	1086	12.3	495	13.0	0.440
Physical activity ^4^							
Sedentary	5798	45.8	4022	45.4	1776	46.7	
Insufficiently active	3354	26.5	2371	26.7	983	25.8	
Active	3515	27.7	2473	27.8	1042	27.4	0.354
Health self-assessment							
Good	10,266	81.1	7191	81.1	3075	80.9	
Regular	2167	17.1	1510	17.0	657	17.3	
Bad	234	1.8	165	1.9	69	1.8	0.930
BMI (kg/m^2^) ^1^	26.3	23.7–29.5	26.3	23.7–29.6	26.2	23.7–29.4	0.939
Waist-to-hip ratio ^1^	0.9	0.8-1.0	0.9	0.8–1.0	0.9	0.8–1.0	0.473
Dyslipidemia ^5^							
No	5237	41.3	3666	41.4	1571	41.3	
Yes	7430	58.7	5200	58.6	2230	58.7	0.985
Hypertension ^6^							
No	8159	64.4	5708	64.4	2451	64.5	
Yes	4508	35.6	3158	35.6	1350	35.5	0.912
Diabetes ^7^							
No	10,634	83.9	7450	84.0	3184	83.8	
Yes	2033	16.1	1416	16.0	617	16.2	0.713
Cardiovascular disease ^8^							
No	12,188	96.2	8529	96.2	3659	96.2	
Yes	479	3.8	337	3.8	142	3.7	0.860

^1^ Median and interquartile range; ^2^ others = Asian and indigenous; ^3^ calculation based on 2009: USD 1.00 = BRL 2.00; ^4^ sedentary: does not perform physical activity; insufficiently active: <150 min/week or exercise < 3 days a week; active: 150 min/week at least 3 days a week; ^5^ LDL ≥ 130 mg/dL or the use of cholesterol reducers; ^6^ systolic blood pressure ≥ 140 mmHg, diastolic blood pressure ≥ 90 mmHg or verified treatment with antihypertensive drugs during the previous two weeks; ^7^ defined as an account of a previous diagnosis of diabetes, the use of medication for diabetes or meeting the diagnostic value of diabetes; ^8^ defined as a report of a heart attack, stroke or revascularization; ^9^ *p* values are derived from Mann–Whitney-tests or chi-square tests.

**Table 2:** Evaluation of prediction accuracy by model, Brazilian Longitudinal Study of Adult Health (ELSA-Brasil), 2008–2010.

	RMSE ^1^	MSE ^2^	MAE ^3^
User-Based CF	1.49	2.21	1.26
Item-Based CF	1.67	2.78	1.40

^1^ Root mean square error; ^2^ mean squared error; ^3^ mean absolute error.

**Table 3:** Confusion matrix by model, Brazilian Longitudinal Study of Adult Health (ELSA-Brasil), 2008–2010.

	**User-Based Collaborative Filtering (UBCF)**							
**K**	**TP**	**FP**	**FN**	**TN**	**Precision**	**Recall**	**TPR**	**FRP**
1	0.88	0.12	9.08	0.92	0.88	0.09	0.09	0.11
3	2.70	0.30	7.25	0.75	0.90	0.27	0.27	0.27
5	4.54	0.46	5.41	0.59	0.91	0.46	0.46	0.43
10	9.11	0.89	0.85	0.15	0.91	0.91	0.91	0.84
	**Item-Based Collaborative Filtering (IBCF)**							
**K**	**TP**	**FP**	**FN**	**TN**	**Precision**	**Recall**	**TPR**	**FRP**
1	0.88	0.12	9.08	0.92	0.88	0.09	0.09	0.12
3	2.66	0.34	7.30	0.70	0.89	0.27	0.27	0.33
5	4.45	0.55	5.51	0.49	0.89	0.45	0.45	0.53
10	9.03	0.97	0.93	0.07	0.90	0.91	0.91	0.93

K: k nearest neighbors; true positives (TP): recommended items with intake; false positives (FP): recommended items that without intake; false negatives (FN): Not recommended items with intake; true negatives (TN): not recommended items without intake; precision: percentage of recommended items with intake; recall: percentage of intake items that have been recommended; true positive rate (TPR): percentage of intake items that have been recommended; false positive rate (FPR): percentage of non-intake items that have been recommended.

## Data Availability

For reasons of confidentiality and privacy of the participants, the data used in this work are not available.

## Supplementary Materials

The following supporting information can be downloaded at: https://www.mdpi.com/article/10.3390/ijerph192214934/s1, Table S1: Food groups and list of items eligible as recommendations, Brazilian Longitudinal Study of Adult Health (ELSA-Brasil), 2008–2010.
