# Supplementary material for: Recommender System Based on Collaborative Filtering for Personalized Dietary Advice: A Cross-Sectional Analysis of the ELSA-Brasil Study
Source: Int J Environ Res Public Health. 2022 Nov 13;19(22):14934. doi: 10.3390/ijerph192214934 (PMC9690822; doi:10.3390/ijerph192214934)
Supplement: Supplementary file 1 [file ijerph-19-14934-s001.zip › ijerph-1989349-supplementary.pdf]

Supplementary Material

Table S1. Food groups and list of items eligible as recommendations, Brazilian Longitudinal Study of Adult Health (ELSA-Brasil), 2008-2010.

	Food groups	Foods	Recommender
1	Refined cereals	White rice, refined grains, corn, and breakfast cereals	
2	Whole cereals	Whole-grain rice, oats, granola, whole cereals, and whole meal bread	X
3	Tubers and roots	Potato (stewed, boiled or mashed), boiled cassava, yam, sweet potato, fried potato, cassava, polenta, sweet potato, farofa (cassava flour with complements), cornmeal cuscus; cassava flour and corn flour	X
4	Breads	Bread, rolls, light bread, pita bread, and simple cake	
5	Confectionery	Sweets, caramels, cereal bar, sweet biscuit with filling, cake with filling, sweet bread, tart, trifle, chocolate bar, bonbons, chocolates, chocolate powder, chocolate drink, cappuccino, milk pudding, milk sweet, mousse, jelly, jam, fruit sweets, honey, syrup, sweet bread, fruit ice lolly, fruit salad with complements, and ice cream	
6	Beans and other legumes	Beans (black, red, white, cowpeas, etc.), lentils, chickpeas and peas	X
7	Oilseeds	Walnuts, cashew nuts, Brazil nuts, peanuts, almonds and pistachio nuts	X
8	Fruits	Avocado, pineapple, banana, persimmon, jack, pine nut, guava; orange, tangerine, apple, pear; papaya, mango, watermelon, melon, strawberry, and fruit salad without complements	X
9	Vegetables	Zucchini, chayote, eggplant, pumpkin, lettuce, garlic, beetroot, broccoli, onion, carrot, chicory, watercress, arugula, raw collards, raw spinach, escarole, braised collards, braised spinach, cauliflower, okra, tomato, runner beans, vegetable soup	X
10	Red meats	Boned beef (steak, minced meat and stewed meat), beef on the bone (rib, oxtail), pork, tripe, liver, offal, and stroganoff	
11	White meats and fish	Boiled chicken, fried chicken, chicken breast, boiled, baked or grilled fish, fried fish, shrimp, shellfish, crab, swimming crab, sardine, and tuna	X

12	Processed meats	Sausage, light cold meats (turkey sausage, turkey breast), ham, mortadella, salami, and bacon	
13	Eggs	Fried egg, omelet, scrambled egg, and boiled and poached egg	
14	High-fat dairy products and milk	Full-cream yogurt, full-cream dairy milk, yellow cheeses, and full-cream cheese spread,	
15	Lower-fat dairy products and milk	Light yogurt, semiskimmed dairy milk, skimmed dairy milk, white cheeses, and light cheese spread	X
16	Oils and fats	Margarine, butter, mayonnaise, and light mayonnaise	
17	Pasta	Macaroni, macaroni dishes, and instant macaroni	
18	Salted snacks	Hotdog, popcorn, fried and baked savories, pizza, and instant soup	
19	Juices and other beverages	Coffee with sugar, coffee with sweetener, coffee with no sugar, mate with sugar, mate with sweetener, sugar-free mate, soy extract, artificial juice with sugar, artificial juice with sweetener, sugar-free artificial juice, industrialized juice with sugar, industrialized juice with sweetener, sugar-free industrialized juice, natural juice with sugar, natural juice with sweetener, sugar-free natural juice, and coconut water	
20	Soft drinks	Soft drinks, and diet/light soft drinks	
21	Alcoholic beverages	Beer, wine, and distilled beverages	
